# From Diagnosis to Decision—Fetal Limb Abnormalities

**DOI:** 10.3390/jcm15020486

**Published:** 2026-01-08

**Authors:** Andreea Florentina Stancioi-Cismaru, Razvan Grigoras Capitanescu, Mihaela-Simona Naidin, Cristian Constantin, Marina Dinu, Florin Burada, Oana Sorina Tica, Mihaela Gheonea, Bianca Catalina Andreiana, Razvan Cosmin Pana, Stefania Tudorache

**Affiliations:** 1 8th Department, Faculty of Medicine, University of Medicine and Pharmacy of Craiova, 200349 Craiova, Romania; andreea.stancioi@umfcv.ro (A.F.S.-C.); marina.dinu@umfcv.ro (M.D.); oanabanica25@yahoo.com (O.S.T.); mihaela.gheonea@umfcv.ro (M.G.); 2 Obstetrics and Gynecology Department, Emergency University County Hospital, 200349 Craiova, Romania; razvancosminpana@yahoo.com; 3 Doctoral School, University of Medicine and Pharmacy of Craiova, 200349 Craiova, Romania; 4 Department of Pharmaceutical Marketing and Management, Faculty of Pharmacy, University of Medicine and Pharmacy of Craiova, 200349 Craiova, Romania; mihaela.subtirelu@yahoo.com; 5 Department of Radiology, County Clinical Emergency Hospital, 200642 Craiova, Romania; cristian.constantin2364@gmail.com; 6 Department of Medical Imaging, University of Medicine and Pharmacy of Craiova, 200638 Craiova, Romania; 7 Laboratory of Human Genomics, University of Medicine and Pharmacy of Craiova, 200638 Craiova, Romania; florin.burada@umfcv.ro; 8 Regional Centre of Medical Genetics Dolj, Emergency Clinical County Hospital Craiova, 200642 Craiova, Romania; 9 Department of Pathology, University of Medicine and Pharmacy of Craiova, 200638 Craiova, Romania; bianca.andreiana@umfcv.ro; 10 Pathological Anatomy Department, Emergency University County Hospital, 200349 Craiova, Romania; 11 2nd Department, Faculty of Nursing and Midwifery, University of Medicine and Pharmacy of Craiova, 200349 Craiova, Romania

**Keywords:** limb abnormalities, prenatal diagnosis, accuracy, operator’s expertise, clubfoot

## Abstract

**Background/Objectives:** Our aim was to investigate the diagnostic accuracy of prenatal ultrasound (US) in fetal limb abnormalities. As a secondary target, we wanted to correlate various predictors for the diagnosis accuracy. **Methods:** We prospectively enrolled cases with routine prenatal US performed in five participating centers. Subsequently, we selected and processed all cases with limb abnormalities: suspected, diagnosed, and missed on the prenatal diagnosis scans. We collected data on the type of anomaly, the US equipment and probes used, the operator’s expertise, the gestational age at the diagnosis, the length of the examination, and the use of US reporting form. SPSS 22.0 software was applied to perform the analyses using non-parametric statistical methods. Associations between categorical variables were evaluated using Fisher’s exact test and Chi-square tests. For correlations between the gestational age and the anomaly severity, we used Spearman’s rank-order correlation. Predictive performance of operator- and scan-related variables for diagnostic accuracy was assessed using receiver operating characteristic (ROC) curve analysis, with area under the curve (AUC) estimates, standard errors (SE), confidence intervals (95% CI), and *p*-values reported. **Results:** Our data showed that most US examinations were performed as part of routine screening (79.7%), and the most frequent anomaly diagnosed was clubfoot. Operators’ expertise demonstrated the highest predictive performance, while technical parameters—scan duration (AUC = 0.20, *p* = 0.1188) and US equipment (AUC = 0.30, *p* = 0.3478)—did not significantly predict diagnostic accuracy. **Conclusions:** The overall diagnostic accuracy of prenatal US was 85.5%. Our findings indicate that diagnostic performance is driven primarily by operator expertise and training, rather than by gestational age at scan and technical parameters.

## 1. Introduction

Fetal limb abnormalities include defects in limb formation (amelia, meromelia), in skeletal patterning (polydactyly, syndactyly, reduction defects), in bone growth/ossification (skeletal dysplasias), and can occur in isolation or can be syndromic.

Recent data show that accurate detection of congenital cardiac and non-cardiac anomalies is feasible in early pregnancy [1,2,3]. Detection rates (DRs), false-negative, and false-positive rates depend on the type of anomaly [4]. The use of a standardized protocol is beneficial [5]. All prenatal findings may have a critical importance in prenatal counseling [6]. As a prenatal diagnostic procedure, US scan requires precision and accuracy and implies efforts to minimize errors and misinterpretations. Adequate training and certification of practitioners help guarantee competency and consistency in interpreting results [7], while proper licensing ensures accountability and adherence to professional and ethical standards [8]. Also, the availability of high-quality equipment plays a role in increasing diagnostic accuracy and DRs [9]. Investigation of predictors for accuracy in prenatal diagnosis is beneficial in interpreting the risk of false-positive and false-negative diagnosis; early parental reassurance may be provided; unnecessary anxiety and interventions may be avoided; also, exploring theses predictors is informative for healthcare systems policy. A multidisciplinary approach, integrating advanced/multimodal imaging and genetic testing, is commendable for optimal outcomes.

Our study aim was to prospectively evaluate the diagnostic accuracy of prenatal US in fetal limb abnormalities. As a secondary target, we investigated which factors can predict an accurate prenatal diagnosis of fetal extremity abnormalities by means of US.

## 2. Materials and Methods

This is a prospective, multicenter observational cohort study. We collected data from 5 prenatal diagnosis units on cases with suspected fetal extremity abnormality on prenatal US scan (index scan). Among these 5 units, ours was included—a tertiary referral center. During the designated study period, 15 examiners were involved in scanning. Their classification in terms of expertise, licensure, and operator-specific volume was established at the beginning of the study. Similarly, the US equipment used was categorized at study initiation. To mitigate the spectrum bias related to the inclusion of a tertiary referral center, we adopted a multicenter design including non-referral units and enrolled consecutive cases.

We enrolled patients between January 2021 and June 2025. The aim was to reach an adequate sample size with available postnatal follow-up (neonatal exam and postnatal imaging in selected cases) for outcome verification. At the beginning of the study, we hypothesized that the six predictors selected would be significant, thus we investigated the following primary predictors of interest:

- Type of anomaly: limb formation—limb reduction, skeletal patterning (polydactyly, syndactyly), position anomaly (clubfoot, clenched hands), long-bones abnormalities in growth/ossification (skeletal dysplasias).
- US equipment (machine generation/probe).
- Operator expertise (years, accreditation, volume).
- Gestational age (GA) at scan (weeks).
- Length of the examination (minutes).
- Use/completeness of US reporting form (completed vs. incomplete).

Consecutive eligible cases meeting inclusion criteria were included.

Inclusion criteria:

- Singleton pregnancies with suspected fetal extremity abnormality on US performed during the study interval (first scan suspecting the limb anomaly or referral scan).
- At least one US prenatal scan performed in the 5 prenatal diagnosis units, regardless of being performed at the end of the first trimester scan (later than 11 weeks), in the second and/or the third trimester.
- Postnatal exam or pathological autopsy available.
- Willingness to provide informed consent for postnatal follow-up.

Exclusion criteria:

- Twin pregnancy.
- Postnatal verification impossible (including lost to follow-up).
- Termination of pregnancy without confirmatory postnatal exam (pathological autopsy not performed or results not available).

We used as reference gold standard the postnatal clinical examination, postnatal radiographic examination, postnatal magnetic resonance (MR)/pathological autopsy performed within 7 days, all confirming the presence and the type of the specific extremity anomaly.

Given that prenatal US report may correctly identify the presence of an extremity anomaly and its type/location, we correlated the data as follows: “accurate”, “partial accurate”, and “missed” in the database. We predefined three categories to classify the diagnostic concordance between prenatal and postnatal findings. We included the case in the “accurate prenatal diagnosis” category if a complete agreement was confirmed postnatally, regarding the presence, type, laterality, and anatomical extent of the limb anomaly. A “partially accurate diagnosis” was defined if the limb abnormality was detected in the prenatal life, but the characterization in the ultrasound form was incomplete, including incorrect laterality, underestimation of anomaly severity, partial description of the affected structures (e.g., detection of a limb reduction defect without the accurate specification of involved bones), or misclassification within the same anomaly group. The diagnosis was considered “missed” if no limb abnormality was identified prenatally and it was diagnosed postnatally

Data collected in the case report form (CRF) fields:

- Patient ID, center, date of scan
- Gestational age at scan (weeks + days)
- Indication for scan (screening, anomaly suspicion/referral)
- Type of suspected anomaly
- Group of suspected anomalies (upper vs. lower extremities)
- Laterality (left/right/bilateral)
- Machine: manufacturer and model, probe frequency, software version, 2D/3D/4D
- Operator: ID, years’ experience, fetal US accreditation, volume of fetal scans
- Length of exam (start time/end time = minutes)
- Measurements taken (which bones)
- Use of form: yes/no, type of form (basic, medium, complex)
- Prenatal diagnosis (pre-coded categories)
- Images stored available (Y/N)
- Follow-up planned (yes/no)
- Additional tests ordered (fetal MRI, karyotype)

Postnatal CRF:

- Date of abortion/birth, gestational age at termination of pregnancy
- Postnatal exam findings (clinical/imaging/autopsy results) coded same categories
- Final classification: “accurate”, “partial accurate”, “missed”
- Suspected reason for discordance

Ethical approval was obtained in each center. Informed consent included permission for image storage and postnatal follow-up.

### Statistical Analysis

Excel was used to establish the database of the risk factors of fetal congenital malformation. SPSS 22.0 software was applied to perform the analyses using non-parametric statistical methods, given the categorical and ordinal nature of the data. Associations between categorical variables were evaluated using Fisher’s exact test and Chi-square tests, including linear-by-linear association where appropriate. Correlations between gestational age and anomaly severity were assessed using Spearman’s rank-order correlation.

Predictive performance of operator- and scan-related variables for diagnostic accuracy was assessed using receiver operating characteristic (ROC) curve analysis, with area under the curve (AUC) estimates, standard errors (SE), confidence intervals (95% CI), and *p*-values reported. A *p*-value of <0.05 was considered statistically significant.

## 3. Results

A total of 98,750 fetuses were scanned during the study period, and 73 cases of limb anomalies were identified. Of these, approximately 5.5% (*n* = 4) were lost to follow-up, resulting in 69 cases that were ultimately included in the final analysis.

### 3.1. Demographics and Examination Characteristics

Most US examinations were performed as part of routine screening (79.7%), with a smaller proportion conducted following anomaly suspicion or referral (20.3%).

**Figure 1:**
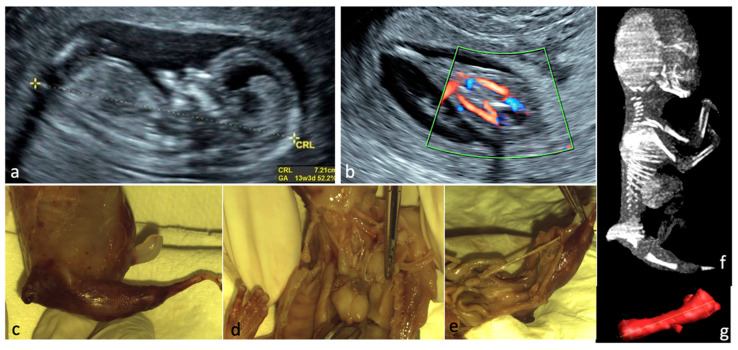
Sirenomelia. (**a**)—Sagittal view of the fetus at FT anomaly scan, showing normal profile, normal nuchal translucency, and present nasal bone. (**b**)—Single lower limb, pathognomonic aspect for sirenomelia, two femoral arteries seen. (**c**–**e**)—Aspects from conventional autopsy: sagittal macroscopic view of the fetal pelvis, frontal view of the heart and great vessels (right lung removed), bilateral absent kidneys. (**f**)—CT scan, sagittal view of the fetus (CT scan performed after obtaining the autopsy data). (**g**)—Single femur measurements, Cone Beam CT technique used.

**Figure 2:**
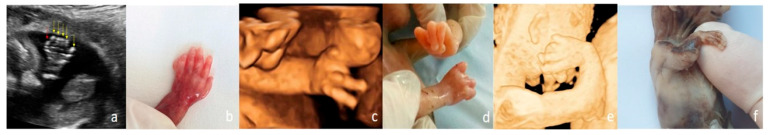
Defects of the upper limbs. (**a**,**b**)—Polydactyly at 16 weeks: US features—the supernumerary finger indicated by the red arrow and postbortum specimen. (**c**,**d**)—Clenched hands in a FADS case (Fetal Akinesia Deformation Sequence); the fetus had normal findings at the FT anomaly scan and developed limbs abnormalities toward the second trimester (US aspect in 3D surface rendering mode and postabortum specimen). (**e**,**f**)—Hypoplasia of the radius and ulna—(US aspect in 3D surface rendering mode and postabortum specimen); simultaneous oligodactyly may be appreciated in both images.

**Figure 3:**
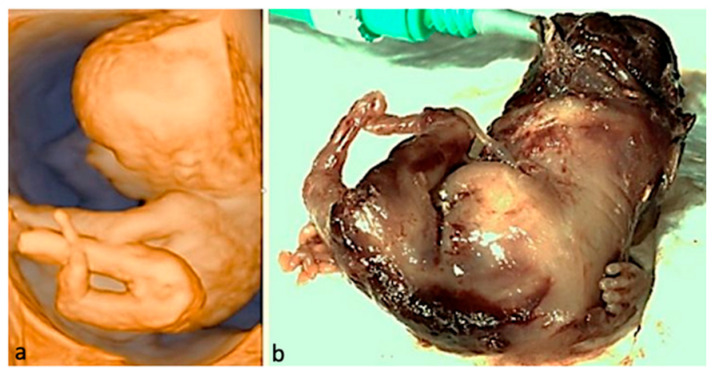
Limb-body wall complex. The abdominal organs in this fetus developed outside the body; the umbilical cord was absent; major limb deformities and severe scoliosis may be seen; given the fatal condition, the parents opted for termination of pregnancy. (**a**)—3D surface rendering applied at FT anomaly scan. (**b**)—postabortum specimen.

**Figure 4:**
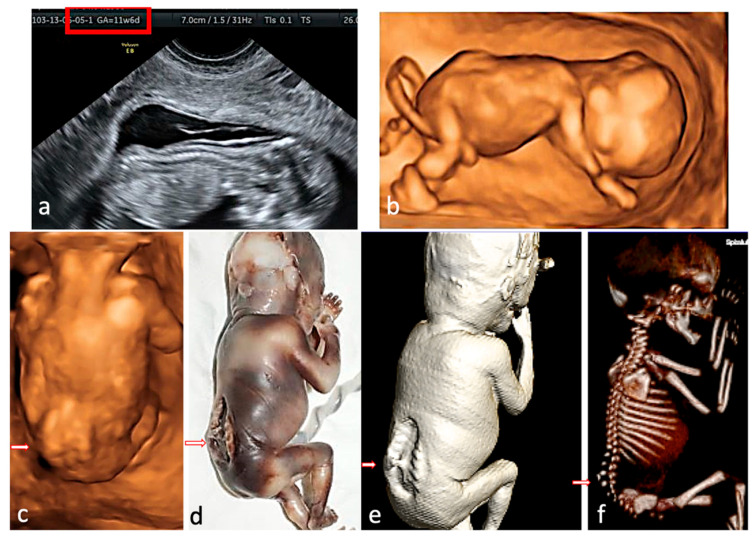
Rachischisis. The fetus presented with lower limb paralysis. (**a**)—2D conventional US, sagittal view of the fetus at FT anomaly scan, showing the lack of regularity of the overlying skin. (**b**,**c**)—3D surface rendering applied at FT anomaly scan: sagittal view of the fetus, showing normal intrafetal ratios, and frontal view from the posterior aspect, suggesting lumbar-sacral scoliosis and additional hemivertebra. (**d**)—postabortum specimen, macroscopic appearance. (**e**,**f**)—CT scan surface rendering, showing the skin defect and confirming the hemivertebra, the level and the extent of the spine anomaly.

**Figure 5:**
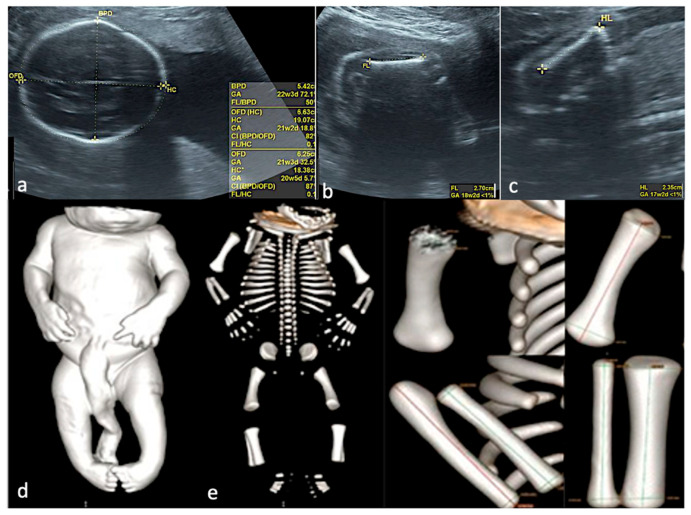
Skeletal dysplasia. (**a**–**c**)—Prenatal US data, showing severe intrafetal disproportions: biparietal diameter—corresponding to 22 weeks and 3 days, femur length—corresponding to 18 weeks and 2 days, humerus length—corresponding to 17 weeks and 2 days. (**d**)—CT scan of the specimen, surface rendering, confirming the severe intrafetal disproportions (very short long bones). (**e**)—CT, Cone Beam CT technique used, allowing each bone measurement.

Regarding the severity of the anomalies, most cases were classified as moderate (55.1%), followed by severe (29.0%) and mild anomalies (15.9%). Laterality analysis showed that anomalies were most commonly bilateral (66.7%), while unilateral involvement was less frequent, with 21.7% affecting the right side and 11.6% the left. Anatomically, anomalies of the lower limbs predominated (58.0%) [Figure 1], followed by the upper limbs (24.6%) [Figure 2] and combined upper and lower limb involvement (17.4%) [Figure 3, Figure 4 and Figure 5].

Almost all examinations were performed using high-end US equipment with 3D/4D capability (92.8%), while only 7.2% used standard 2D devices. More than half of the scans were performed by senior operators with more than 10 years of expertise (59.4%), followed by intermediate-level (29.0%) and junior-level (11.6%) examiners. A similar distribution was observed in operator licensing, with most operators being fetal medicine specialists (MFM, 59.4%), followed by obstetricians/gynecologists (39.1%), and one general practitioner (1.4%).

Slightly more than half of examinations lasted 15–30 min (55.1%), while 43.5% extended beyond 30 min and only 1.4% were completed in under 15 min. Follow-up assessments were planned in 40.6% of cases, and genetic testing was recommended in an identical proportion (40.6%).

**Figure 6:**
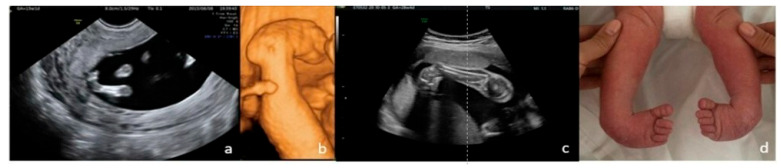
Bilateral clubfoot. (**a**)—2D FT anomaly scan, showing the abnormal angle of the left foot (both feet seemed rotated inward). (**b**)—3D surface rendering applied at ST anomaly scan, confirming the clubfoot suspicion. (**c**)—2D third-trimester image of the right foot (both feet rotated inward). (**d**)—postnatal aspect.

**Figure 7:**
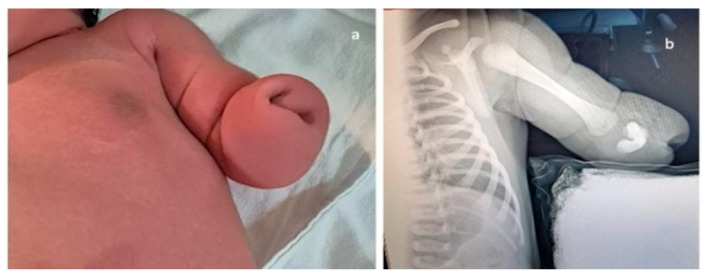
Upper-limb unilateral (left) reduction defect. In this case, of transverse limb deficiency, the defect was diagnosed at the end of the first trimester. Complete genetic work-up was performed, rendering normal results; the follow-up scans strengthened the suspicion of isolated anomaly, and the parents decided to continue pregnancy. (**a**)—postnatal aspect. (**b**)—radiography confirming the presence of the well-developed humerus and rudimentary buds of the radius and ulna. Multidisciplinary counseling was offered repeatedly, and led to surgical reconstruction and prosthetics, with good functional results.

Postnatal diagnoses confirmed that the most frequent anomaly was clubfoot (40.58%) [Figure 6], followed by limb reduction defects (20.29%) [Figure 7], polydactyly (13.04%), shortened/angulated bones (10.15%), joint contracture/abnormal position (10.15%), others (4.35%) and syndactyly (1.44%).

**Figure 8:**
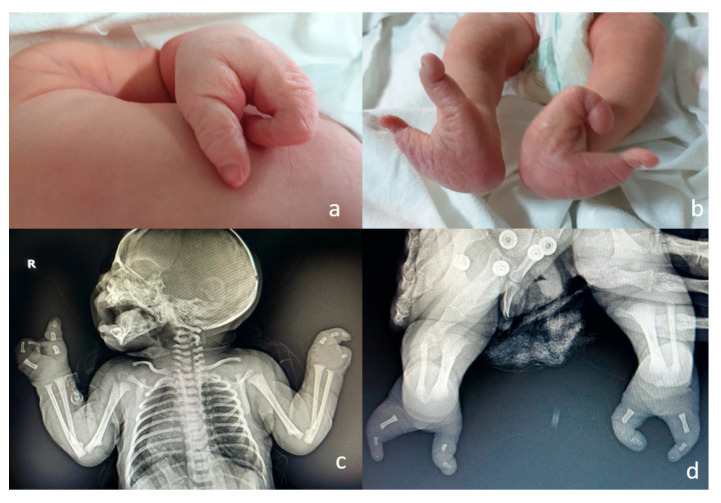
Ectrodactyly—this case was missed in the prenatal period. A single third-trimester scan was performed, at 36 weeks of amenorrhea. (**a**,**b**)—postnatal aspect: split hand and split foot malformation; the absence/defect of the central fingers and toes may be appreciated, giving the “lobster claw” appearance. (**c**,**d**)—postnatal CT, confirming the clinical aspect.

The overall diagnostic accuracy of prenatal US was 85.5%, while 14.5% of anomalies were either missed or only partially accurate compared with postnatal findings [Figure 8].

### 3.2. Concordance Between Prenatal and Postnatal Diagnosis

Prenatal detection of clubfoot, polydactyly, and syndactyly demonstrated perfect diagnostic performance across all metrics (100%), indicating excellent reliability. Limb reduction defects exhibited slightly lower sensitivity (79%), suggesting that some cases were missed or misclassified, though specificity and PPV remained high. The “Other” category showed high sensitivity and NPV, but slightly reduced specificity and PPV, reflecting the inherent variability and complexity in classifying less common or ambiguous anomalies [Table 1].

These findings reinforce the overall diagnostic strength of prenatal US for identifying structural limb anomalies, while also highlighting areas—particularly rare or complex deformities—where diagnostic accuracy may be improved through enhanced imaging protocols, referral for expert examination or multidisciplinary assessment.

Table 1 summarizes the diagnostic performance metrics of prenatal US for various limb anomalies. The sensitivity, specificity, positive predictive value (PPV), and negative predictive value (NPV) were calculated for each US feature/type of anomaly, based on concordance with postnatal diagnoses.

### 3.3. Association Between Operator Expertise and Diagnostic Accuracy

**Figure 9:**
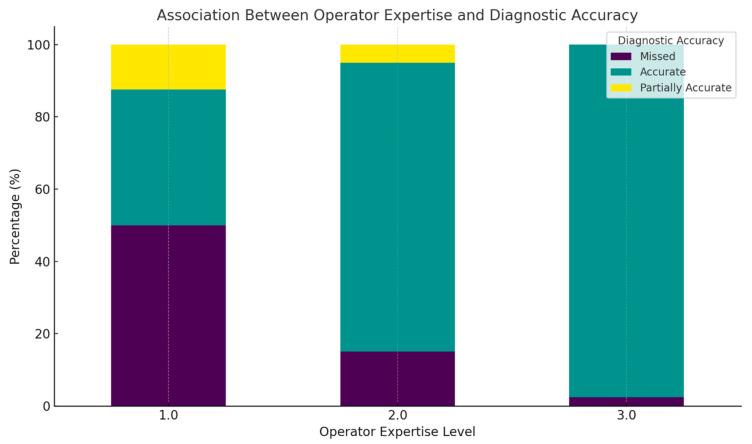
This figure displays the percentage distribution of prenatal diagnostic accuracy across different operator expertise levels using a stacked bar chart. The diagnostic outcomes are color-coded as follows: missed diagnoses are represented in dark purple, accurate diagnoses in green, and partially accurate diagnoses in yellow.

A strong association is observed between operator expertise and diagnostic accuracy [Figure 9].

At Expertise Level 1, the largest portion of the bar is dark purple, indicating that approximately half of the cases were missed. The remaining cases are split between accurate (green) and partially accurate (yellow) diagnoses, suggesting significant diagnostic variability among less experienced operators

For Expertise Level 2, the teal segment becomes predominant, showing that over 80% of the cases were accurately diagnosed, with reduced dark purple and yellow segments. This reflects improved performance with moderate experience.

At Expertise Level 3, the bar is almost entirely teal, indicating nearly 100% diagnostic accuracy, with virtually no missed (purple) or partially accurate (yellow) diagnoses. This emphasizes the role of high-level operator expertise in achieving reliable prenatal anomaly detection.

The color-coded visualization clearly illustrates a progressive improvement in diagnostic accuracy with increased operator experience. These findings reinforce the necessity of expert-level training and oversight in fetal US practice, particularly for limb anomaly screening. In clinical settings, prioritizing evaluations by highly trained sonographers may significantly reduce misdiagnosis rates and enhance prenatal care outcomes.

### 3.4. Correlation Between Gestational Age and Anomaly Severity

Spearman’s rank-order correlation was used to assess the relationship between GA at examination and the severity of the diagnosed anomaly [7].

The analysis revealed a weak, negative correlation (ρ = −0.167, *p* = 0.197), which was not statistically significant.

**Figure 10:**
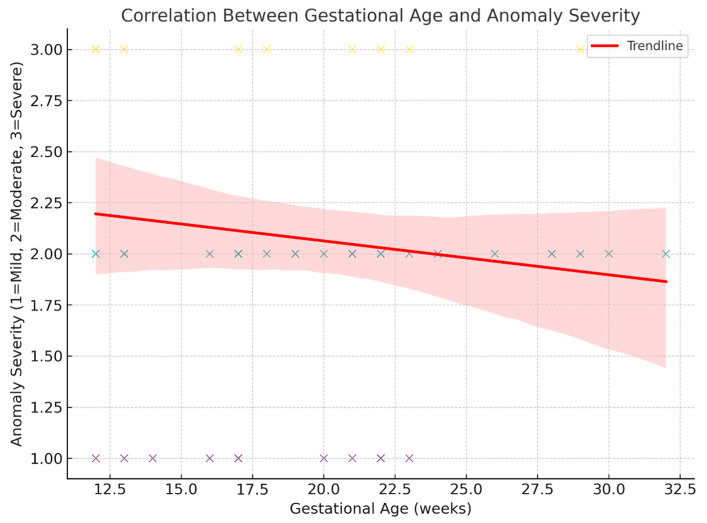
Correlation between GA at examination and the severity of the diagnosed anomaly.

**Figure 11:**
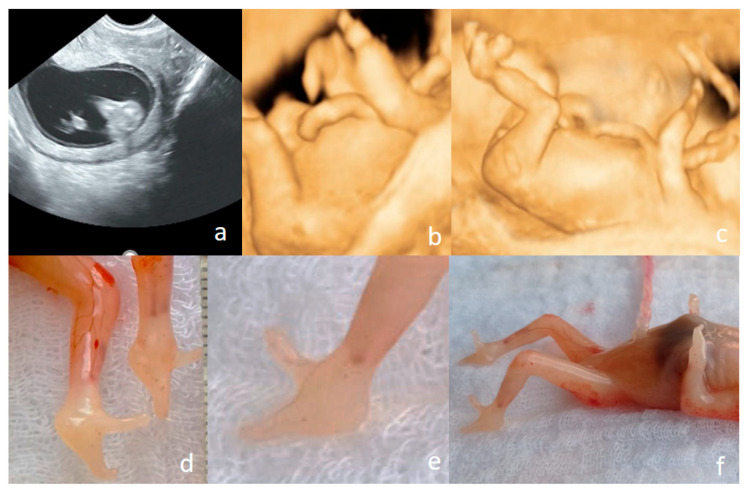
Ectrodactyly diagnosed at the FT anomaly scan (12 weeks and 1 day) ((**a**–**c**)—US features, (**d**–**f**)—postabortum aspects). (**a**)—bilateral cleft foot, seen in 2D conventional US. (**b**)—upper limb presented in 3D US surface rendering showing that the terminal portions of the limbs end in one to two rudimentary digit-like projections, lacking normal phalangeal segmentation; no normal thumb–finger visible; both left and right upper limbs show nearly identical abnormalities. (**c**)—split foot malformation; the absence/defect of the central fingers and toes may be appreciated, giving the “lobster claw” appearance. (**d**–**f**)—postabortum aspects—split foot malformation (the “lobster claw” appearance) in feet may be seen; the four-limb involvement may be appreciated.

This suggests that anomaly severity was not meaningfully associated with GA in this dataset, although the trend indicates slightly more severe anomalies that were seen at earlier gestational ages [Figure 10 and Figure 11].

### 3.5. ROC Curve Analysis

The variable with the highest predictive value was Operator Expertise (AUC = 0.90), indicating that clinicians with greater experience were substantially more likely to achieve the correct diagnoses. US Form Completion Level also showed strong discriminative power (AUC = 0.85), suggesting that systematic and thorough documentation correlates with diagnostic accuracy. For ROC curve analysis, AUC estimates and 95% confidence intervals were calculated using nonparametric methods. Given the limited sample size for certain predictors, confidence intervals occasionally extended beyond the theoretical range of 0–1; these values were truncated to the valid range for interpretative purposes. Type of Anomaly showed moderate predictive capacity (AUC = 0.69), which may reflect the inherent variability in detectability across different anomaly types. In contrast, Scan Duration (AUC = 0.20) and US Equipment Type (AUC = 0.30) demonstrated poor predictive value, implying that these technical factors alone do not substantially influence diagnostic performance. Given the exploratory nature of the ROC analyses and the limited number of outcome events, AUC results should be interpreted as hypothesis-generating rather than definitive.

**Figure 12:**
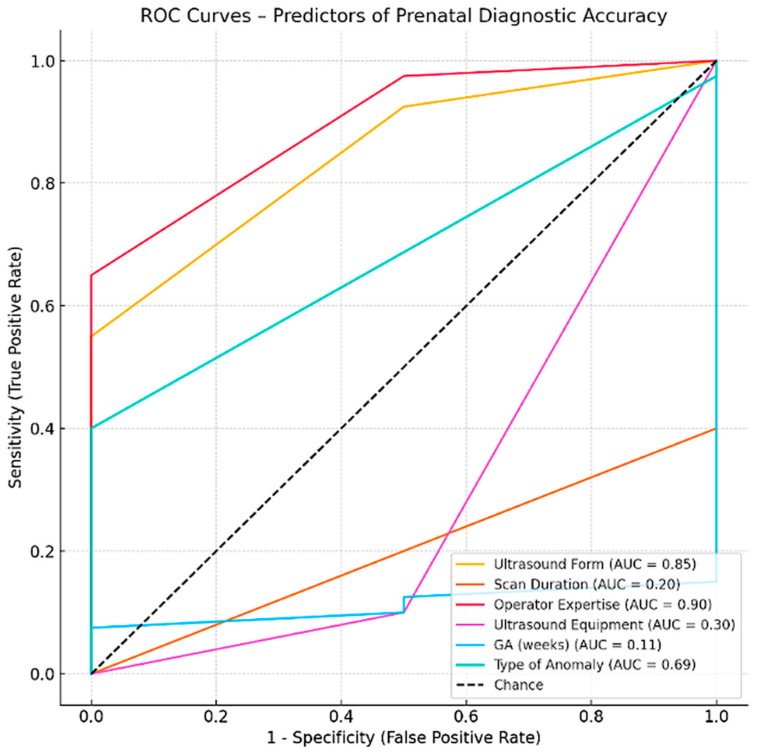
ROC Curves—Predictors of Prenatal Diagnostic Accuracy.

The gestational age at the time of scan (GA in weeks) was not retained in the final AUC summary due to data structure limitations, though its role should not be entirely discounted in clinical interpretation [Figure 12].

A significant association was found between operator expertise and the accuracy of prenatal US diagnosis. The Chi-square test revealed that diagnostic accuracy varied significantly across expertise levels (χ^2^(4) = 20.30, *p* = 0.0004). Additionally, Spearman’s rank correlation indicated a moderate positive relationship between expertise and diagnostic performance (ρ = 0.48, *p* < 0.001). These findings suggest that operators with greater clinical experience are more likely to provide accurate diagnoses. This emphasizes the impact of human factors—such as training and experience—on the effectiveness of prenatal imaging [Table 2].

## 4. Discussion

The prevalence of limb abnormalities is approximately 6 in 10,000 live births [10].

Studies from European registries and published literature on routine prenatal screening show wide variation in the prenatal DRs of fetal limb abnormalities. The figures are extremely low in isolated abnormalities (e.g., 8.3% for longitudinal LRDs and 0 for split hand/foot) [11], but accuracy may reach very high values in dedicated and performance settings (up to 77.8%) [12]. This confirms significant regional and institutional differences. The wide differences in DRs reported reflect varying criteria for definition of malformation, the performance of methods used for postnatal examination, the selection of the study population, the prevalence of specific associated conditions, and—most probably—the operators’ expertise levels [4,12,13].

DRs in limb abnormalities vary significantly depending on the specific type of limb abnormality. Data suggests that while routine US can detect most major limb abnormalities involving long bones and joint contractures, it misses a substantial proportion of minor defects (polydactyly, other digital abnormalities). As expected, most studies show that major limb defects are easier to detect than minor ones (long bone defects (76%) vs. digit-only defects (29%). The reported sensitivity for US is 19.1% for polydactyly, 76.0% for abnormal hand position, and 76.0% for limb reduction defects involving the long bones, and 81.3% for arthrogryposis [14]. In the mentioned recent systematic review and metanalysis, the overall DR in prenatal life was 77.8%. Although major limb abnormalities (ectrodactyly, sirenomelia) and limb absence can be suspected in very early pregnancy (9–10 weeks) [4], the diagnosis of polydactyly is limited (15% before 22 weeks) [14].

In our study, most US examinations were performed as part of routine screening (79.7% of cases), with a smaller proportion conducted following anomaly suspicion or referral (20.3%). We reached an overall diagnostic accuracy of prenatal US of 85.5%—reasonably higher than in most large studies. A total 14.5% of anomalies were either missed or only partially accurate compared with postnatal findings. We hypothesize that this difference may be attributed to several factors. We used high-end US equipment with 3D/4D capability (in 92.8% of cases), while in only 7.2% standard 2D devices were used. Bones, having a straight and regular surface from the US physics perspective—they have a high acoustic impedance, high reflection, and an acoustic shadowing phenomenon. Thus, bones allow us to confirm their presence and shape by giving a high contrast between them and surrounding tissues. From the embryological point of view, bones ossify early enough to be seen clearly at the end of the first trimester. Moreover, bones are ideally seen in 3D US reconstruction images, and most machines nowadays have included the required software for interpretation. Finally, we used, for many years, a detailed protocol in the leading center in this study, the US form including confirmation of the three segments for the four limbs in the FT and digits in the ST. If a certain feature is not obtained at the routine scan, the study internal policy made mandatory reexamination and/or referral, at any given gestational age. In our view, these factors (the physics of the system studied, the modern equipment used, and the intern protocol) led to the high figures in accuracy.

Postnatal clinical, imaging, and pathological examinations were performed as part of routine care. While postnatal assessors were not blinded to prenatal findings, the postnatal diagnosis was based on objective reference standards: clinical examination, radiography, CT, MRI, and/or autopsy.

Our analysis demonstrated a non-significant negative correlation between anomaly severity and the GA at diagnosis, suggesting that the fetal size at scan did not play a decisive role in the timing of anomaly detection. Although the negative direction of the coefficient may indicate a trend toward more severe anomalies being identified earlier in gestation, this association did not reach statistical significance. Notably, limb anomalies encompass a wide spectrum of severity, anatomical complexity, and sonographic visibility—from subtle distal defects to major structural abnormalities— which can significantly influence their detectability, regardless of the GA at scan. Therefore, diagnostic accuracy and timing are likely more dependent on the intrinsic characteristics of the anomaly and its US appearance, rather than GA alone. Furthermore, standardized screening protocols and examiner expertise are likely to have a significant impact on detection timing.

Regarding anomaly severity, most cases were classified in our study as moderate (55.1%).

Out of the 69 diagnosed cases, 24 pregnancies were terminated. Among these, 13 were classified as having severe limb anomalies, 5 terminations were performed at the end of the first trimester upon maternal request in accordance with current legislation, and 6 cases presented with additional major extra-skeletal anomalies (complex brain malformations, omphalocele, cystic hygroma, megacystis, left heart hypoplasia with transposition of the great arteries and right renal agenesis, and Tetralogy of Fallot, respectively). It is important to highlight that 13 pregnancies were terminated on the basis of extremity abnormalities only. This underlines the importance of an accurate diagnosis in prenatal life, and we plead for a cautious, gentle, and multidisciplinary counseling process. In isolated defects, parents should be informed of all postnatal options and management strategies available, from reconstructive surgeries utilizing lengthening to prosthetic integration. These innovative approaches aim to restore limb function and enhance the quality of life for affected individuals.

The reported incidence is higher in the upper limbs compared with the lower limbs [15]. Contrary, in our study, anomalies of the lower limbs predominated (58.0%), followed by the upper limbs (24.6%), and combined upper and lower limb involvement (17.4%).

According to previous studies [16], unilateral defects are more common than bilateral defects and there is a right-sided predominance in fetal limb abnormalities. In our case series, limb anomalies were most commonly bilateral (66.7%), while unilateral involvement was less frequent, with 21.7% affecting the right side and 11.6% the left.

In our study, the postnatal assessment confirmed that the most frequent anomaly detected was clubfoot (40.6%), followed by limb reduction defects (15.9%), and polydactyly (13.0%). A total of nine cases were missed prenatally: one case of osteogenesis imperfecta, four cases of limb reduction defects, three cases of clubfoot, and one case of genu recurvatum. The missed diagnoses can be attributed to a combination of technical, operator-related, and patient-related factors. The operator’s expertise was very high in the first case, yet he failed in the prenatal detection of these subtle and evolving musculoskeletal abnormalities (a slightly curved and normal-length femur was seen in the late third-trimester images). Unfavorable fetal position and equipment limitations were involved in all cases with missed limb reduction defects. In this group, oligohydramnios and increased maternal body mass index also impaired sonographic assessment in two cases. Lack of adequate prenatal care, with third-trimester booking, was found in three cases among the four. In the group of missed clubfoot, we failed to find an obvious explanation in two cases, and we also found late booking in one. It is worthwhile mentioning that during retrospective assessment of the ultrasound files, the team of authors easily spotted the clubfoot in both second-trimester scanned cases. In this group, all operators used limited ultrasound forms. The genu recurvatum case was missed in the context of severe oligohydramnios, footling breech presentation, and late second-trimester booking. These findings highlight the multifactorial nature of missed prenatal diagnoses and underscore the importance of optimal scanning conditions, adequate operator training, and comprehensive prenatal follow-up to improve diagnostic accuracy.

Our study results suggest that the length of the US examination was not significantly influenced by the operator’s level of training, as both junior and senior operators had comparable scan times.

The data we obtained confirm that operators’ skills is one of the critical factors in successful prenatal detection [8].

Lower levels of detection would be expected in smaller centers and in operators with less volume in prenatal diagnosis scanning. In our study, accuracy also increased with experience, from 37.5% (95% CI: 9–76%) among junior operators (<5 years) to 85.0% (95% CI: 62–96%) among intermediate-level operators (5–10 years), and 97.6% (95% CI: 87–100%) among senior operators (>10 years).

Operator experience and expertise represent the most modifiable factor in improving prenatal DRs of limb abnormalities, having the potential to improve outcome and counseling through specialized training.

Among the studied predictors, the operator expertise demonstrated the highest predictive performance (AUC = 0.900, *p* < 0.001). In contrast, technical parameters such as scan duration (AUC = 0.200, *p* = 0.1188) and US equipment (AUC = 0.300, *p* = 0.3478) did not significantly predict diagnostic accuracy. The GA at examination showed no predictive value (AUC = 0.1125, *p* = 0.0129).

As for the advantages of our study, it should be mentioned that this is a rather large, prospective study, enrolling 69 patients from an unselected cohort of approximately 115,000 fetuses scanned, and lasting for 4.5 years. We involved five prenatal diagnosis units, among them—a tertiary fetal medicine center—to increase generalizability and operator variability. Involving a tertiary referral center, we are aware that we may face a spectrum bias (usually, referral centers see more complex cases). This is the second reason for multicenter recruitment. In terms of originality, we used imaging postabortum and postpartum to complete information after pregnancy termination.

As for limitations, the postnatal examiners were not blinded to the prenatal report. We did not review a random sample from stored images, to assess inter-observer variability and to validate reporting accuracy. We had a 5.5% loss to follow-up and unavailable postnatal confirmation cases, and this figure may impair statistical analysis in accuracy studies. Confounding factors (persistent unfavorable fetal position, oligohydramnios, high BMI, unmeasured operator skill features) were not studied. Another limitation of the present study is that the inter-observer agreement analysis was not performed; the prenatal image interpretation was conducted by a single operator *per case* and no independent blinded re-reading was undertaken.

As for practical considerations, our study supports several conclusions: continuous training and using a uniform, standardized, previously agreed upon US form seem beneficial. Continuum in training of doctors involved in prenatal diagnosis is crucial to ensure accurate interpretation of results, minimize errors, and provide safe guidance for expectant parents. Skilled practitioners not only improve diagnostic accuracy but also allow appropriate counseling. Operators should use a checklist of required views for fetal extremities: four limbs, all long bones—in at least two different views. Sonographers should attempt to obtain images of hands, feet and digits at each anomaly scan—if the equipment and the time interval allocated for the examination allow this attempt. This has the potential to reduce variability.

In our view, future research directions should be considered: quantifying the independent effects of documentation on long term (possibly the volume of stored information), establishing the optimal duration of an examination, the role of integrating AI-assisted imaging in routine practice, and the real impact of standardized forms across diverse clinical settings. Understanding the independent impact of structured US documentation (stored images and/or video clips) could clarify whether form completion itself, beyond protocol content, improves DRs and standardizes care. AI may reduce operator dependence and increase sensitivity, especially for subtle or distal anomalies, but real-world effectiveness needs validation. Establishing the optimal duration of an examination could improve efficiency and ensure high-quality care across diverse clinical settings.

## 5. Conclusions

High-quality US remains the cornerstone of diagnosis in fetal extremity abnormalities, supplemented by 3D imaging, MR, and CT. Proper characterization, detection of associated anomalies, and multidisciplinary counseling are crucial for prognosis and management.

The most important predictors of accurate prenatal diagnosis of fetal extremity abnormalities are operators experience and expertise. The type and the extent of the anomaly, the gestational age, the equipment quality, and examination length play supporting roles. The role of high-volume documentation, although it seems helpful, is yet to be proved.

While significant progress has been made, the spectrum bias cannot be avoided. Efforts should be dedicated to improve professional training and licensing, and to centralizing cases if required, as the most important predictor for prenatal detection is also the most modifiable one.

Further research is needed to optimize protocols, leverage new technologies, and ensure consistent, high-quality prenatal diagnosis of fetal extremity abnormalities.

## Figures and Tables

**Table 1:** Diagnostic Performance of Prenatal US by Anomaly Type.

Anomaly Type	Sensitivity	Specificity	PPV	NPV
Normal	0.0	0.87	0.0	1.0
Clubfoot	0.9	0.98	0.96	0.93
Polydactyly	1.0	1.0	1.0	1.0
Syndactyly	1.0	1.0	1.0	1.0
Limb Reduction Defect	0.64	0.95	0.7	0.93
Shortened/Angulated Bones	0.26	1.0	1.0	0.78
Joint Contracture/Abnormal Position	0.0	0.93	0.0	1.0
Other	0.0	0.96	0.0	1.0

**Table 2:** The analysis of the six predictors in the accuracy of prenatal US diagnosis.

Variable	AUC	SE	95% CI	*p*-Value	Interpretation
US Form	0.8500	0.1021	0.65–1.05	0.0006	Excellent predictor
Scan Duration	0.2000	0.1924	−0.18–0.58	0.1188	No discriminative power
Operator Expertise	0.9000	0.0751	0.75–1.05	0.0000	Excellent predictor
US Equipment	0.3000	0.2130	−0.12–0.72	0.3478	No discriminative power
GA (weeks)	0.1125	0.1558	−0.19–0.42	0.0129	No discriminative power
Type of Anomaly	0.6875	0.1695	0.36–1.02	0.2685	Fair predictor

## Data Availability

All ultrasound files, neonates’ files, and autopsy files are available and will be provided upon reasonable request.
